# Simulation-Rooted Assessment of Electromagnetic Calorimeters Based on Silicon Sensors

**DOI:** 10.3390/s25164918

**Published:** 2025-08-09

**Authors:** Petru Mihai Potlog, Veta Ghenescu, Alina Tania Neagu

**Affiliations:** Institute of Space Science, Subsidiary of INFLPR, 409 Atomistilor Street, 077125 Magurele, Ilfov, Romania

**Keywords:** electromagnetic calorimeters, Geant4 simulations, detector design, silicon sensors, tungsten absorbers

## Abstract

We present a Geant4-based simulation study of the electromagnetic sampling calorimeter (ECAL) foreseen in the LUXE experiment. The ECAL will enable precise measurement of the number and energy spectrum of positrons and electrons. The electromagnetic shower response, energy resolution, and linearity—properties that are essential for physics research—are studied. The Geant4 simulation model provides a good description of the data from the literature, and the stochastic energy resolution is comparable to the state-of-the-art resolution for a Si-W calorimeter. The simulated ECAL model consists of layers of solid-state sensors interspersed between tungsten plates, with the sensors divided into pads. The advantage of these detectors is their high active layer density, which facilitates the construction of more compact devices. A detailed description of the sensor response using MC simulations is fundamental to detector design and predicting energy measurement performance. We collected simulated data using electron beams in the energy range of 2 to 18 GeV, with a step of 2 GeV. The signal size distribution measured in the test beam campaign is well reproduced by the Geant4 simulation, confirming the accuracy of the simulation approach. The analysis described in this paper focuses on electromagnetic shower reconstruction and characterizes the ECAL response to electrons in terms of energy resolution and linearity.

## 1. Introduction

The simulation of particle generation during initial collisions, their interaction with detector materials, and the resulting detector response has become increasingly crucial in recent experiments. This importance has grown with each new generation of experiments, from SLD [1], LEP, and B-factories, progressing through the Tevatron [2], and continuing to the present-day experiments at the LHC [3]. This trend is due to the higher precision requirements of these experiments. Such simulations will continue to play a significant role in upcoming experiments, including those at super-B factories [4], the International Linear Collider (ILC) [5], the Future Circular Collider (FCC) [6], as well as in numerous ongoing and forthcoming smaller-scale experiments. Multipurpose detectors have been employed in particle physics investigations today, mostly at colliders, with the goal of acquiring as much accurate and comprehensive data as possible for the reconstruction of every particle in the final state of every collision—including its type and kinematic characteristics. Trackers use the curvature of ionization trails in magnetic fields to deduce the momenta of charged particles, while calorimeters only indirectly determine particle energies by looking at the debris that emerges from the particles’ contact with a block of matter, so-called showers. The reconstruction of jets, which comprise a variety of particle types, is incomplete without calorimeters.

**Figure 1:**
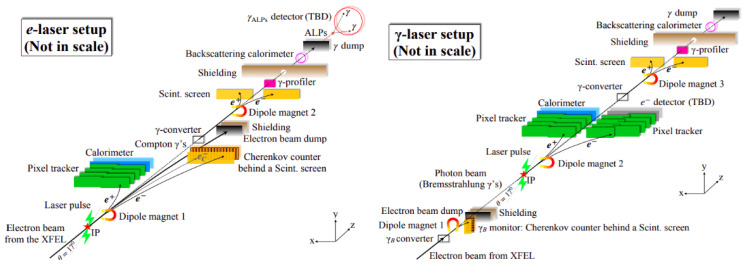
Schematic layouts for the *e-laser* (**left**) and *γ-laser* (**right**) setups (from [7]).

The LUXE [7] (Laser Und XFEL Experiment) aims to explore strong-field quantum electrodynamics (QED) and search for new physics beyond the Standard Model by colliding high-energy electrons with intense laser pulses. The proposed LUXE experiment foresees the use of a 16.5 GeV electron beam from European XFEL [8] and a high-power laser beam of up to 350 TW. Two configurations are envisioned for the forthcoming LUXE experiment, called *e-laser* and *γ-laser* setups, with their detailed—not to scale—layouts represented schematically in Figure 1.

Various detectors optimized for the expected fluxes of particles detect the electrons and positrons produced in these collisions. A silicon pixel tracking detector and a high-granularity calorimeter detect the positrons, while a scintillation screen and gas Cherenkov detectors measure the electrons. As a key experimental setup component, the electromagnetic sampling calorimeter (ECAL) is designed to precisely measure the number and energy spectrum of positrons and electrons produced in these interactions.

To support the detector development, we performed a detailed simulation study of the ECAL using the Geant4 toolkit [9]. Extensive Monte Carlo (MC) simulations will provide substantial insight into the ECAL characteristics which will contribute to optimizing the electromagnetic calorimeter design. Moreover, simulating the silicon sensor response is fundamental for this endeavor. This simulation serves as a starting point for detector design and is an indispensable tool for predicting the detector’s performance.

The analysis presented here focuses on extended configuration of the calorimeter, which consists of 20 layers of 3.5 mm thick tungsten absorbers interleaved with 320 µm thick silicon pad sensors. This configuration corresponds to a total depth of 20 radiation lengths and is optimized for high granularity and compactness.

The main objective is to evaluate the ECAL’s response to electrons over a wide energy range (2.0 to 18.0 GeV) using Monte Carlo simulations. Key performance indicators such as energy deposition, linearity, and energy resolution are analyzed. The proposed configuration is expected to achieve a high degree of linearity and a stochastic energy resolution comparable to state-of-the-art silicon–tungsten (Si–W) calorimeters.

This study confirms the suitability of the calorimeter geometry for the LUXE experiment and highlights the reliability of Geant4-based simulations in reproducing key detector performance metrics. Incident electrons with an energy in the range from 2.0 to 18.0 GeV were shot perpendicularly on the ECAL surface and the obtained results are presented. Section 2 and Section 3 present the details of the sampling electromagnetic calorimeter geometry and the Geant4 simulation package, respectively. Section 4 discusses the results obtained. The last section outlines the main conclusions of this study.

## 2. Detector Design

**Figure 2:**
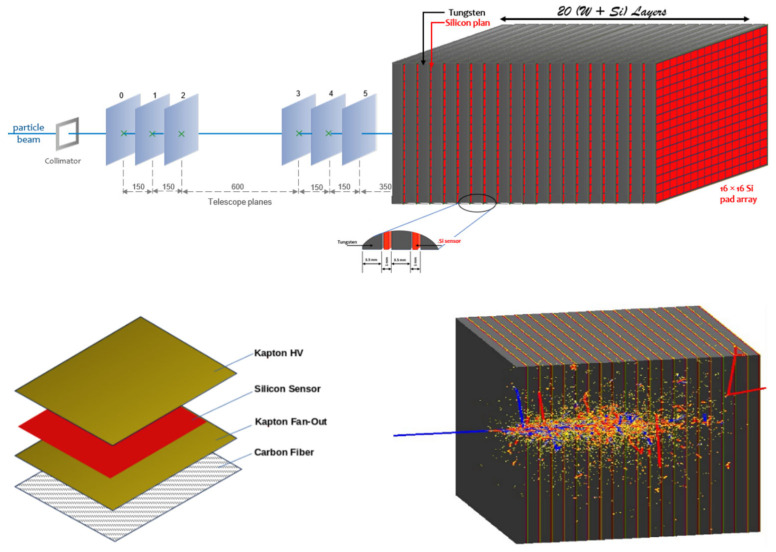
Experimental setup implemented in Geant4 (**up**) with the sensor assembly details (**bottom left**) and Geant4 visualization of 10 GeV electrons interaction on ECAL (**bottom right**).

The electromagnetic calorimeter (ECAL) developed for the LUXE experiment is designed to measure the energy and multiplicity of high-energy electrons and positrons emerging from laser–electron interactions. The calorimeter is a sampling type, optimized for compactness, linearity, and high spatial and energy resolution, which are essential characteristics given the constrained detector space and the demanding precision requirements of the experiment. The calorimeter consists of 20 layers of 3.5 mm thick tungsten absorbers, each corresponding to approximately one radiation length (X_0_), interleaved with 320 μm thick silicon sensors. The total depth of the calorimeter is therefore 20 radiation lengths, providing sufficient material to fully contain electromagnetic showers in the energy range of interest (2.0–18.0 GeV). The tungsten plates are separated by 1 mm gaps, into which the silicon sensors are inserted as active layers. The fine segmentation of the sensors allows for high spatial resolution in reconstructing the longitudinal and transverse shower development. While the full ECAL for LUXE will consist of an array of silicon sensors covering a larger area (56 × 9 cm^2^), the present study focuses on a reduced prototype version, using a single 9 × 9 cm^2^ sensor per layer. Each of these sensors is segmented into 16 × 16 individual pads of 5 × 5 mm^2^ active area, providing spatial granularity for shower reconstruction. Figure 2 (up) presents the Geant4 layout of the simulated ECAL configuration, with the setup used in the test beam campaigns. The bottom left side provides a close-up view of the sensor assembly with the Kapton HV. Kapton Fan-Out, Carbon Fiber—used for sensor support—and the silicon sensor itself, segmented into 5 × 5 mm^2^ pads. The bottom right side shows the simulated development of a 10 GeV electromagnetic shower fully contained in the calorimeter; this visualization confirms that the selected geometry is adequate for full shower containment within the detector volume.

Silicon sensors are employed as the active material due to their well-established performance in high-energy physics applications [10]. It has been extensively used in radiation detector systems due to its mature fabrication technology, cost-effectiveness, and reliable performance under radiation exposure. These characteristics make it particularly suitable for the LUXE experiment, where sensors must maintain stable and reliable performance in an environment subject to intense particle fluxes. A comparison of key physical properties of silicon with other semiconductor materials used in particle detectors is provided in Table 1.

Although alternatives such as germanium (Ge) and gallium arsenide (GaAs) offer certain advantages—such as higher electron mobility—silicon remains the preferred choice due to its availability, robustness, and wide adoption in detector technology.

## 3. MC Simulation

Monte Carlo simulations of the ECAL response and the electromagnetic shower evolution are performed using a standalone detector simulation application based on Geant4 (version 11.2.2) and ROOT (version 6.34.02) software [18]. All simulation steps, from the generation of the incident particle beam to the production of the ECAL response can be performed within this application. The detailed ECAL detector geometry described in Section 2 is defined (*module DetectorConstruction*) by specifying the type, size, material, and position of the detector volumes. The tungsten absorber plates and the active layers are implemented as solid boxes with various characteristics. The Si active sensor per layer is implemented as 320 μm thick Si with a matrix of 16 × 16 pads, whereas the pad size is 5 × 5 mm^2^. Particle transport through the whole detector geometry as well as the deposition of charge carriers in the active sensor volumes is performed by the *DetectorHit* and *EventAction* modules, while for the physics list we used *FTFP_BERT_EMZ* option. This physics list includes all relevant electromagnetic processes for the study—such as electron ionization, bremsstrahlung, and multiple scattering—and employs a selection of the most accurate models from the low-energy and standard electromagnetic physics packages in Geant4. The physics list was chosen for its enhanced precision in thin layers, particularly relevant for finely segmented silicon sensors like those in the ECAL. All particles are tracked through the full detector volume, with energy deposition recorded in each sensitive pad. Simulated electron beams were injected perpendicular to the calorimeter surface, targeting the center of the sensor plane, and each run consisted of 500,000 primary electrons. The electrons’ energy is sampled from a Gaussian distribution with an energy spread of 50 MeV. The design choice of Geant4 is based on object-oriented methodology and C++ language to provide modular and flexible software. All physical processes, models, and visualization modules are fully accessible to the user. Its object-oriented design allows users to implement or modify any physics process in Geant4 without the need to alter other software components. We used this MC package to evaluate the simulation response of a sampling electromagnetic calorimeter for various electron energies, leveraging its specific features. The ECAL configuration, along with all its materials and settings, was converted into C++ language and simulated using the Geant4 Monte Carlo code. All geometry, physics lists, and particle sources can be easily modified or extended. The user has full access to physics models and visualization modules, which were also used to generate figures such as the electromagnetic shower development in Figure 2.

## 4. Results

It is known that when a high-energy electron, positron, or photon collides on a thick absorber, it initiates an electromagnetic cascade [19] as pair production, bremsstrahlung, and Compton scattering effects generate electrons/positrons, and photons of lower energy. Due to the significant number of particles (including electrons, positrons, and photons) involved in a high-energy electromagnetic cascade, the researchers looked for general parameters to characterize typical shower behavior. Scaling variables, such as the radiation length X_0_—the fundamental unit of length for electromagnetic processes—can be used to quantify radiation effects. We use a method that measures the number of charged particles produced in each electromagnetic shower to measure the energy of incident electrons. Electrons produce an electromagnetic shower, and the resulting shower profile and energy deposited in each active layer contribute to the optimization of the calorimeter granularity as well as to the study of possible transverse and longitudinal energy leakage. Analytic descriptions are certainly valuable, but in the high-energy domain, the necessity arises to employ statistical methodologies, specifically Monte Carlo simulations, for meticulous step-by-step reproduction of the processes governing the formation of electromagnetic showers.

The simulated data for the silicon sensors in the ECAL have been previously validated against experimental test-beam measurements, as shown in ref [20]. This comparison has shown excellent agreement between simulation and data, confirming the reliability of the simulation framework. Therefore, the results presented here for the calorimeter geometry described in Section 3 can be trusted to accurately reflect the expected detector performance.

### 4.1. Energy Response and Linearity

**Figure 3:**
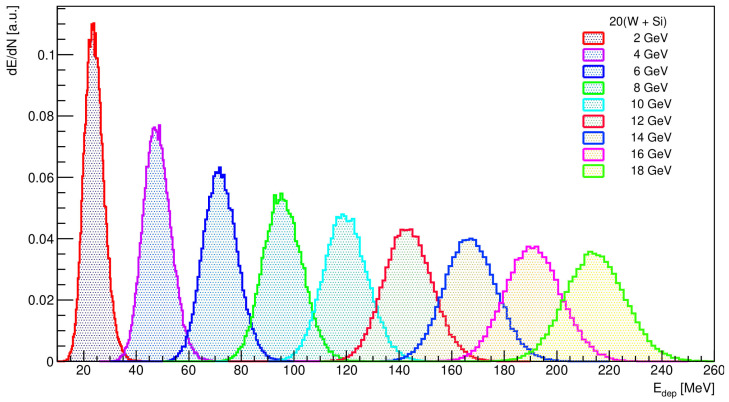
The measured energy spectra of 2–18 GeV electron events collected in the central region for a detector configuration expressed in arbitrary units.

As indicated above, in high-energy physics, one of the state-of-the-art solution for reliable simulations is the Geant4 framework. The developed Geant4 application simulates the passage of electrons through the successive tungsten and sensor planes and records the energy deposited in the specific geometrical volume designated as a sensitive detector. The energy of the incident electron is determined through the process of quantifying the number of charged particles (*N*) present within each electromagnetic shower. Figure 3 shows the distribution of the energy deposition in the calorimeter, dE/dN expressed in arbitrary units, for electron incident energies of 2 GeV to 18 GeV, registered at every 2 GeV.

**Figure 4:**
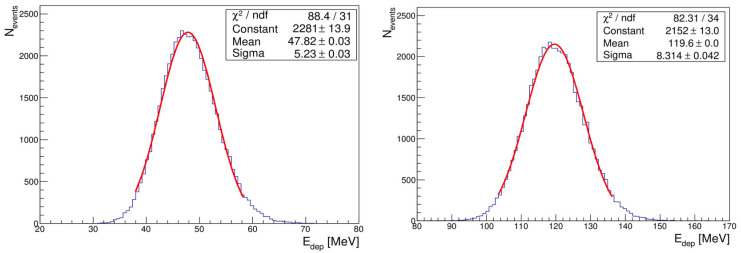
Distribution of the total energy deposition of 4 GeV (**left**) and 10 GeV (**right**) electron beam fitted with a Gaussian.

The distributions show Gaussian peak structures with the most probable values increasing with energy as expected. The energy deposition data considered for inclusion in the analysis are limited to values within two standard deviations (2σ) of the mean value. This selection criterion is used to ensure that the analysis includes data points located within the central, statistically representative region of the distribution, thereby mitigating the influence of outliers. In the case of an ideal calorimeter, the observed response is directly proportional to the energy of the incoming particle. These distributions were fitted with a Gaussian function whose mean *μ* and standard deviation *σ* are taken and given in Table 2. Two examples of the energy deposited distribution for 4 GeV and 10 GeV electron beam fitted with a Gaussian function are shown in Figure 4.

**Figure 5:**
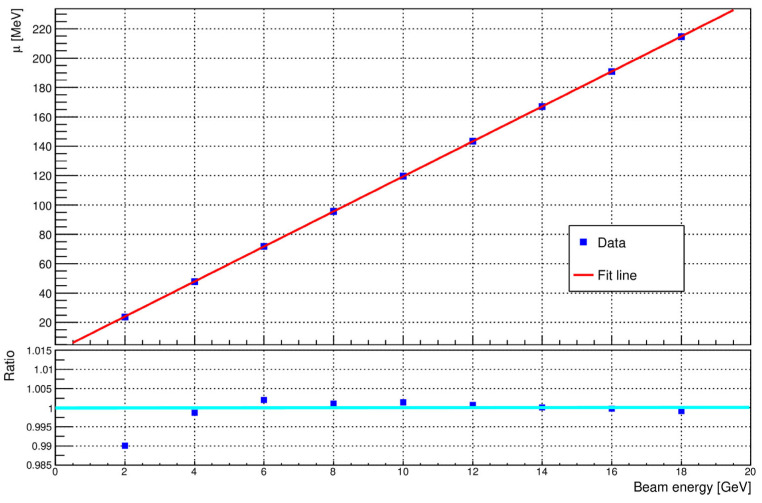
Mean energy detected by the calorimeter, *μ*, as a function of beam energy. The red line shows the linear fit of the simulation data. The lower part of the figure shows the ratio of the deposited energy to the straight-line fit.

The results show that the deposited energy rises proportionally with the beam energy while the spread remains relatively low. The energy response was found to be linear to better than 1% over the full range. Figure 5 shows the mean energy deposited in the calorimeter as a function of the incoming energy.

The full line is a least square fit to the data:

$$E_{\mathrm{dep}}=\left(11.92\pm 0.01\right)E+\left(0.18\pm 0.11\right)\left[\mathrm{MeV}\right]$$

where $E_{\mathrm{dep}}$ is the detected energy in MeV and E is the incoming electron energy in GeV. This confirms that the proposed calorimeter geometry provides a stable and linear energy response suitable for precise energy measurements.

### 4.2. Longitudinal Shower

**Figure 6:**
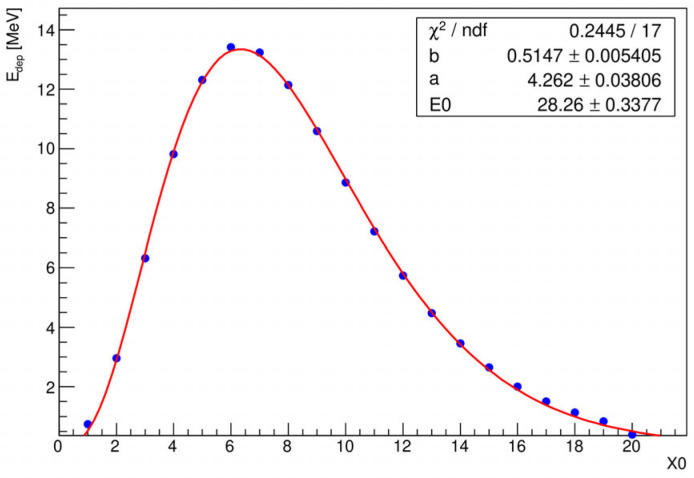
Longitudinal shower development vs. the depth in number of radiation lengths for 10 GeV incoming electrons fitted with function from Equation (1).

The longitudinal profile represents the average response of ECAL per layer, with each layer equivalent to a thickness of 1 *X*_0_ in the z-direction, and reflects how the electromagnetic shower evolves as it progresses through the material. Figure 6 shows the longitudinal shower profile from the Monte Carlo simulation for 10 GeV incident electrons. The fact that average longitudinal shower profiles can be accurately described by a gamma distribution is widely recognized [21], as outlined in Equation (1):

(1) $$F\left(E,t\right){=E}_{0}b\frac{{\left(\mathrm{bt}\right)}^{a-1}e^{-\mathrm{bt}}}{\Gamma \left(a\right)}$$

The depth of the maximum, *t*_max_, can be calculated from the shape parameter *a* and the scaling parameter *b* according to the following:

(2) $$t_{\max}=\frac{a-1}{b}$$

Table 3 shows a comprehensive summary of the expected maximum shower depth for the various energies of the incident electrons and the specific calorimeter geometry implemented in Geant4 application.

**Figure 7:**
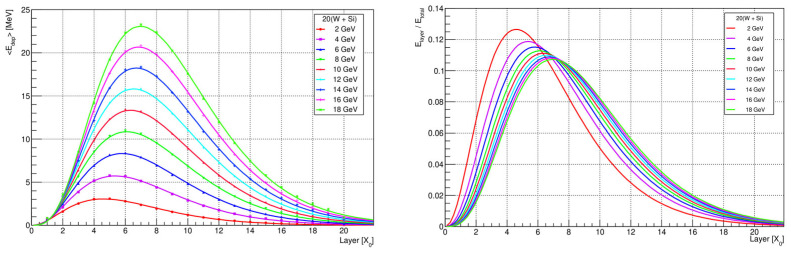
Comparison of longitudinal shower development (**left**) and normalized longitudinal shower (**right**) vs. the depth in number of radiation lengths for 2, 4, 6, 8, 10, 12, 14, 16, and 18 GeV.

Figure 7 shows the longitudinal profiles (i.e., the distribution of the average energy deposited in each calorimeter layer as a function of the layer number) for all considered electron energies. The detector’s total depth of 20 *X*_0_ is sufficient to fully contain the electromagnetic shower, even for the highest-energy electrons (18 GeV), with only a negligible fraction of the energy (well below 0.5%) reaching the final layer. The calorimeter layers provide uniform sampling of the shower development along its entire length. For low energies (e.g., 2 GeV), the shower maximum occurs around the 5th layer, and the signal is measured symmetrically around this point. For higher energies (e.g., 18 GeV), the maximum shifts deeper, near the 7th layer, so the initial layers primarily record the rising part of the shower profile. As a result, the distribution of energy deposition across the layers shifts with the beam energy, which is important for characterizing the calorimeter response and calibrating it for precise energy reconstruction over a broad energy range.

### 4.3. Energy Resolution

Energy resolution is a key performance parameter used to characterize the response of a calorimeter. The relative energy resolution of a calorimeter [22], $\sigma_{E}/E$, can be expressed as a cumulative result of various contributions. The behavior as a function of energy is parametrized by a fit of the form ~$1/\sqrt{E_{0}}$, leading to the following:

(3) $$\frac{\sigma_{E}}{E}=\frac{a}{\sqrt{E}}⊕\frac{b}{E}\text{⊕}c$$

where a is the stochastic term, b represents the contributions due to electronic noise, and c is a constant term such as non-uniformity or imperfect calibration. The “⊕“ symbol indicates a quadratic sum. The stochastic term takes into account the statistical fluctuations in the shower detection, and it is larger for sampling calorimeters, but its effect decreases with growing energy.

**Figure 8:**
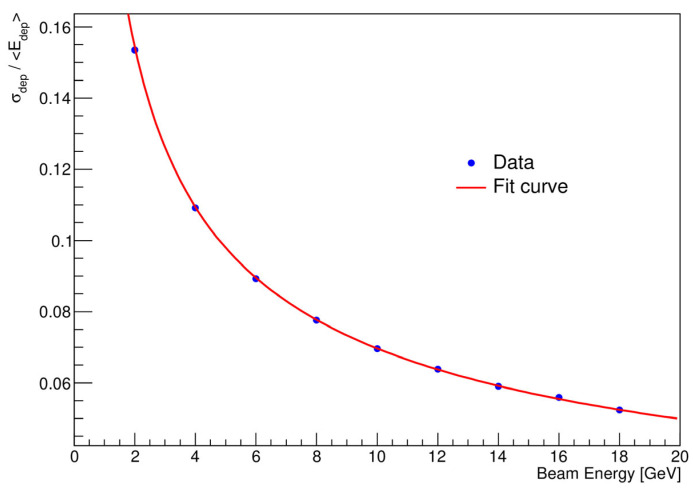
Energy resolution of the simulated calorimeter prototype as a function of electron energy. The red line is a fit with Equation (3).

In sampling calorimeters, the energy deposited in the active medium fluctuates event-by-event because the active layers are interleaved with absorber layers. These fluctuations represent the most important limitation to the energy resolution of these detectors and are due to variations in the number of charged particles that cross the active layers. The second term contributing to energy resolution arises from electronic noise within the readout chain and is contingent on the detector technique and the characteristics of the readout circuit, including detector capacitance and cables. The third term consists of contributions that are independent of the energy of the particle. The resolution for electromagnetic showers with the ECAL is shown in Figure 8. In this figure, the values of $\sigma_{E}/E$ are shown as a function of E, together with the fit using Equation (3).

From fitting Equation (3) to the data in Figure 8, a stochastic energy resolution term of 0.2168 ± 0.0003 was extracted. In addition, the following parameters were obtained from the fit: b = 0.0 ± 0.0114 and c = 0.0119 ± 0.0007. The constant term, c, accounts usually for imperfections in the calorimeter’s construction and is sensitive to any inconsistencies in the detector’s response; but in our case, these errors are not worth too much consideration. Maintaining a low constant term (approximately 1%) ensures good energy resolution at high energies. The results obtained—see Figure 8—demonstrate the excellent performance of the ECAL.

## 5. Conclusions

The Geant4 simulation toolkit is a cornerstone in the field of nuclear and particle physics, particularly in the area of the detectors used to measure electrons, positrons, and photons. Its widespread adoption underlines its importance in research efforts. Key to the success of the Geant4 is the continued validation of its physics models, along with research and development efforts aimed at improving computational efficiency. Achieving meaningful validation in such scenarios demands close collaboration between Geant4 developers and experimental groups to craft simulations that accurately reflect real-world conditions. In this paper, Monte Carlo studies using electrons of 2 to 18 GeV energy have been performed to optimize the segmentation of the silicon–tungsten calorimeter. The goals were to extract the energy resolution $\sigma_{E}/E$ and the longitudinal shower profile shape for the implemented ECAL. The analysis of energy deposition distributions provides profound insights into the calorimeter’s performance across varying electron beam energies. The calorimeter response is linear to within approximately 1%, as shown in Figure 5. This highlights the detector’s remarkable capacity to precisely capture and quantify the energy of incoming particles, with an evident improvement in accuracy as energy levels increase. First exploratory studies of the shape of electromagnetic showers have also been performed. Longitudinal profiles have been obtained, reproducing the expected global features of the evolution of an electromagnetic shower. The detector’s depth adequately accommodates most of the shower, even for high-energy electrons (18 GeV), with only a minor portion of the energy (less than 0.5%) being deposited in the final sensor layer.

The simulation has been shown to accurately reproduce all relevant measurements, including the mean reconstructed energy, energy deposition distributions, and longitudinal energy profiles.

Furthermore, the dependence of the measured energy resolution on the incident beam energy E can be characterized as $\sigma_{E}/E$ $\sigma_{E}/E=21.68/\sqrt{E\left(\mathrm{GeV}\right)}⊕1.19\%$. These results are comparable to those obtained by the CALICE collaboration for a similar silicon–tungsten calorimeter, underlining the robustness of the design and the accuracy of the simulation. Overall, this work highlights the effectiveness of the Geant4 simulation toolkit in modeling the electromagnetic shower development and calorimeter response, supporting its role as a vital tool for detector development and performance studies in particle physics.

## Figures and Tables

**Table 1:** Comparison of different semiconductor materials [11,12,13,14,15,16,17].

Properties/Material	Si	Ge	GaAs
Atomic Number	14	32	32
Band Gap [eV]	1.12	0.66	1.43
Density [g/cm^3^]	2.33	5.32	5.32
Mean E to create an e^−^ h^+^ pair [eV]	3.62	2.9	4.2
Electron mobility [cm^2^/Vs]	1500	3900	8500

**Table 2:** Mean *μ* and standard deviation *σ* values for electron energies between 2 and 18 GeV.

E [GeV]	µ [MeV]	σ [MeV]
2	23.79	3.65
4	47.82	5.22
6	71.87	6.42
8	95.67	7.42
10	119.58	8.32
12	143.37	9.16
14	167.13	9.87
16	190.92	10.67
18	214.61	11.25

**Table 3:** Maximum shower depths at different electron energies.

*E* [GeV]	*t_max_* [X_0_]
2	4.602
4	5.350
6	5.788
8	6.095
10	6.338
12	6.532
14	6.700
16	6.844
18	6.969

## Data Availability

The data presented in this study are available on request from the corresponding author.
